# Astrocyte Heterogeneity in Multiple Sclerosis: Current Understanding and Technical Challenges

**DOI:** 10.3389/fncel.2021.726479

**Published:** 2021-08-11

**Authors:** Chih Hung Lo, Mario Skarica, Mohammad Mansoor, Shaan Bhandarkar, Steven Toro, David Pitt

**Affiliations:** ^1^Department of Neurology, Yale School of Medicine, New Haven, CT, United States; ^2^Department of Neuroscience, Yale School of Medicine, New Haven, CT, United States

**Keywords:** astrocytes, multiple sclerosis, single nucleus sequencing, multiplexed imaging, experimental autoimmune encephalomyelitis

## Abstract

The emergence of single cell technologies provides the opportunity to characterize complex immune/central nervous system cell assemblies in multiple sclerosis (MS) and to study their cell population structures, network activation and dynamics at unprecedented depths. In this review, we summarize the current knowledge of astrocyte subpopulations in MS tissue and discuss the challenges associated with resolving astrocyte heterogeneity with single-nucleus RNA-sequencing (snRNA-seq). We further discuss multiplexed imaging techniques as tools for defining population clusters within a spatial context. Finally, we will provide an outlook on how these technologies may aid in answering unresolved questions in MS, such as the glial phenotypes that drive MS progression and/or neuropathological differences between different clinical MS subtypes.

## Introduction

Due to the resounding success of current MS medications in treating relapsing-remitting MS (RRMS), research interest is increasingly focused on disease progression and neurorepair where current treatments are ineffective. In contrast to RRMS, progressive disease is thought be driven by central nervous system (CNS)-intrinsic processes, where infiltration with peripheral immune cells plays only a minor role. Thus, the attention in MS research is arguably shifting from the peripheral immune system to CNS cells. The CNS-intrinsic processes associated with progression include chronic, diffuse activation of glia cells at the rim of chronic active lesions and in the normal appearing white matter (NAWM) (Chen et al., 2014; Absinta et al., 2019; Sucksdorff et al., 2020), cortical demyelination and neuro-axonal degeneration (Dutta et al., 2006). Identifying the glial subpopulations that contribute to MS progression is likely to yield new targets for therapeutic intervention in progressive MS (Ponath et al., 2018b; Wilbanks et al., 2019; Guerrero and Sicotte, 2020).

In this review, we are focusing on astrocytes. Astrocytes are substantially more numerous than microglia cells, accounting for 20–40% of the total glial population, depending on the brain region (von Bartheld et al., 2016). In a homeostatic state, astrocytes play critical roles in brain function, including synapse formation and elimination (Lee et al., 2021), establishing and maintaining network circuitry, control of neurotransmitters release and uptake (Anderson and Swanson, 2000), modulation of blood-brain barrier (BBB) function (Horng et al., 2017) and blood flow, as well as maintenance of ion and water homeostasis among others (Sofroniew and Vinters, 2009; Ponath et al., 2018b).

CNS pathologies such as trauma, infection, neurodegeneration, and inflammatory demyelination lead to prominent astrocyte responses with morphological, molecular, and functional remodeling. Reactive astrocytes can promote tissue survival by forming a functional barrier around damaged tissue and contribute to tissue repair (Faulkner et al., 2004; Tyzack et al., 2014; Anderson et al., 2016). However, reactive astrocytes may also become dysfunctional and adopt disease-promoting phenotypes. In MS, this includes recruitment and activation of peripheral immune cells and resident microglia (Ponath et al., 2018a), and potentially direct neurotoxic effects on oligodendrocytes and neurons (Liddelow et al., 2017). In addition, reactive astrocytes have reduced capabilities for glutamate uptake (Pitt et al., 2000; Fang et al., 2012) and other homeostatic functions (Schirmer et al., 2019), which may result in indirect neurotoxicity.

Here, we review the progress made in identifying astrocyte populations in MS and its mouse model, experimental autoimmune encephalomyelitis (EAE). We will discuss the limitations of current snRNA-seq methods in identifying astrocyte subpopulations and the potential of highly multiplexed imaging in further elucidating astrocyte heterogeneity by adding spatial information.

## Heterogeneity of Resting Astrocytes

In contrast to oligodendrocytes and microglia, astrocytes are morphologically heterogenous at a resting state. At least nine morphologically distinct astrocyte subpopulations have been identified, of which four subtypes are present within the human neocortex (protoplasmic, radial cells, marginal glia, and perivascular glia) (Matyash and Kettenmann, 2010). Additionally, astrocytes are highly diverse in their functional properties, including calcium dynamics, gap junction coupling, expression of transmitter receptors, membrane currents, and glutamate transporters (Matyash and Kettenmann, 2010).

One of the first efforts to identify astrocyte subpopulations was based on marker expression. Genetically green fluorescent protein (GFP)-labeled astrocytes were isolated and screened for 81 cell surface antigens with fluorescence-activated cell sorting (FACS) (John Lin et al., 2017). This identified five distinct astrocyte subpopulations present across cortex, brainstem, and olfactory bulb, which were subsequently demonstrated to emerge at different developmental stages and to have distinct gene-enrichment and functional properties.

Since their selection as the “Method of the Year 2013” by Nature Methods (2014), single-nucleus and single-cell RNA sequencing (scRNA-seq) have become routine technologies to identify cellular subpopulations based on transcriptome-wide gene expression at single-cell resolution. A study that employed this approach identified astrocyte subtypes in mouse cortex using scRNA-seq and single-cell spatial transcriptomics. By applying multiplexed single-molecule fluorescence *in situ* hybridization (smFISH), the study found that cortical astrocytes segregated into a superficial, mid and deep layer pattern, which did not match the neuronal layering (Bayraktar et al., 2020). However, the spatial clusters did not align with astrocyte clustering based on single-cell transcriptomics, indicating that single cell genomics and smFISH produce different patterns of astrocyte molecular diversity, potentially due to difference in parametric depth.

A separate scRNA-seq study that used Smart-seq2 followed by unsupervised clustering distinguished five distinct astrocyte subtypes in adult mouse cortex and hippocampus (Batiuk et al., 2020). The authors noted that the population structure of astrocytes differed from that of neurons, by lacking distinct cellular hierarchies, suggesting multiple axes of heterogeneity. The transcriptomic differences suggest astrocyte subtype specialization across major astrocyte functions, including synaptogenesis, phagocytosis, synapse function/plasticity, neurotransmission, and others. In addition, the subpopulations were shown to be differentially distributed across the cortex and hippocampus and exhibited distinct morphologies and differential Ca^2+^ signaling (Batiuk et al., 2020).

Together, the single-cell studies provide evidence for transcriptomic heterogeneity of astrocytes, albeit with less distinct cellular hierarchies than neuronal subtypes. The transcriptomic clusters correspond to distinct morphological features and spatial distribution and have implications for different physiological functions. These studies have focused on cortical astrocytes and less is known about astrocyte diversity in white matter (Köhler et al., 2021).

## Heterogeneity of Reactive Astrocytes in EAE and MS

A hallmark of virtually all CNS pathologies is prominent astrocyte responses. The contribution of reactive astrocytes to a given CNS disease is complex and likely driven by multiple concurrent astrocyte phenotypes that vary with disease, disease stage, brain region, age and genetic predisposition (Ponath et al., 2018a; Escartin et al., 2021). Therefore, defining the population structures of reactive astrocytes in different CNS conditions will provide critical insight into their pathogenesis.

In EAE, earlier studies determined astrocytic gene expression in different neuroanatomic regions of mice using a Ribo-tagging approach in transgenic animals, which allows isolation of mRNA from astrocytes only (Itoh et al., 2018). This demonstrated regional differences in astrocytes transcriptomes, including differential expression of complement and cholesterol synthesis pathway genes in astrocytes isolated from spinal cord, optic nerve, cerebellum, hippocampus, and cerebral cortex, consistent with heterogeneity of astrocyte reactivity across different CNS regions.

In an early transcriptome study, astrocyte responses were stratified into neurotoxic and neuroprotective phenotypes termed A1 and A2 (Liddelow et al., 2017), in analogy with the now abandoned concept of M1/M2 (macrophages) and Th1/Th2 (lymphocytes) polarization (Ransohoff, 2016). The A1 phenotype exhibited functional deficits affecting phagocytosis and synapse formation, and was toxic to neurons and oligodendrocytes *in vitro*. A1 phenotypes were found to be present in neurodegenerative diseases and MS, based on the expression of complement component 3, whereas A2 astrocytes, which are characterized by upregulation of several neurotrophic factors, were prevalent in ischemia. This binary classification was initially received with considerable enthusiasm; however, in subsequent studies, only subsets or a mix of A1 and A2 signature genes were found to be upregulated in human diseases and mouse models of acute and chronic CNS injury (Al-Dalahmah et al., 2020; Das et al., 2020; Zhou et al., 2020). A recent consensus statement recommended to move beyond the A1–A2 labels, as they do not capture the functional diversity of reactive astrocytes, and to use multidimensional data to establish distinctive astrocyte phenotypes (Escartin et al., 2021).

A recent study by Quintana et al. that used scRNA-seq and epigenetic analyses in combination with *in vivo* CRISPR-Cas9-based genome editing, identified seven astrocyte subpopulations in EAE (Wheeler et al., 2020). The dominant cluster was characterized by activation of pro-inflammatory and neurotoxic pathways such as the unfolded protein response, activation of NF-κB and inducible nitric oxide synthase pathways. This cluster was driven by downregulation of genes targeted by transcription factor *NRF2*, which limit oxidative stress and inflammation (Wheeler et al., 2020). Moreover, this population was stabilized by epigenetic modifications driven by MAFG and MAT2α signaling, further supporting the notion that epigenetic changes to the CNS contribute to MS pathogenesis (Huynh et al., 2014). In MS lesion tissue, astrocytes were demonstrated to have increased expression of MAFG and decreased expression of NRF2. Recently, the same group identified an anti-inflammatory subset of astrocytes in EAE that was characterized by co-expression of the lysosomal protein, LAMP1, and the death receptor ligand, TRAIL (Sanmarco et al., 2021). This astrocyte population limits inflammation by inducing T cell apoptosis and is driven by IFNγ, produced by natural killer cells within the meninges. A similar population of astrocytes characterized by IFNγ and TRAIL signaling was identified in human brain and this population was reduced by over 80% in MS tissue (Sanmarco et al., 2021).

## Challenges of Determining Astrocyte Heterogeneity With snRNA-seq

Astrocytes can be isolated intact from fresh murine CNS tissue, although enzymatic dissociation has been shown to induce early activation genes (Lacar et al., 2016). In adult human CNS, the method of choice for single-cell transcriptomics is snRNA-seq, because the high cellular interconnectivity makes it difficult to isolate astrocytes and neurons in intact form. snRNA-seq has the additional advantages that it avoids perturbation of gene expression by enzymatic dissociation during whole cell isolation, and that it can be applied to archival frozen material from biobanks (Ding et al., 2020).

Acceptance of snRNA-seq was initially limited, because of differences in RNA quantity and quality between nucleus and cytosol. It was subsequently established that nuclei can be confidently matched to their representing cells, that transcriptomes from single nuclei and whole cells correlate highly, albeit with an abundance of nascent transcripts in nuclei (Lake et al., 2017; Bakken et al., 2018). A recent comparison of snRNA-seq and scRNA-seq data from human microglia demonstrated that a high number of genes implicated in microglia activation in Alzheimer’s disease (AD) was substantially reduced in nuclei (Thrupp et al., 2020). This highlights that single-nucleus data are highly sparse and noisy, particularly in glial cells, requiring improved methodologies for recovery of biological signals (see below).

An additional problem of snRNA-seq studies is that the ratios between different CNS cell types differ to those determined by neuro-histological cell quantification (von Bartheld et al., 2016). The neuroanatomic literature suggests that glia cells are approximately 1.5–2 times more numerous than neurons in cortical gray matter, and 3–4 times more numerous in combined cortex and white matter. Oligodendrocytes are the most frequent glial cell type (45–75%), followed by astrocytes (19–40%), and microglia (10%), depending on the brain region (von Bartheld et al., 2016).

In snRNA-seq data, human brain astrocytes and microglia are consistently under-represented. In several landmark studies of human adult cortex, astrocytes constituted between 1.9 and 7.2%, and microglia between <1 and 2.1% of total nuclei (Lake et al., 2018; Zhu et al., 2018; Hodge et al., 2019). In the two snRNA-seq studies of MS white matter lesion tissue, the predominant nuclear populations were neurons, oligodendrocytes and oligodendrocyte precursor cells constituting 80% of total CNS nuclei, whereas astrocyte nuclei accounted for 7 and 11% and microglia nuclei 2.4 and 2.9%, respectively (Jäkel et al., 2019; Schirmer et al., 2019). A possible reason for this underrepresentation is that they are preferentially lost during the preparation steps presumably due to different mechanical properties of astrocyte/microglia nuclei. Moreover, neurons express up to 10 times more RNA than glial cells, which leads to overrepresentation of neuronal RNA in the final library composition (Zeisel et al., 2015). Collectively, this leads to low representation of astrocyte/microglia nuclei, reduced library complexity and consequently low resolution of population structure heterogeneity for glial cells.

In two recent studies, Quintana et al. performed scRNA-seq of astrocytes derived from fresh, rapid autopsy materials of MS patients using 10x Genomics v2.0 chemistry (Wheeler et al., 2020). This data set was integrated with previously published snRNA-seq results of astrocytes in MS and healthy brains. The authors used this approach to confirm the presence of a specific astrocyte population in MS, which was previously identified in EAE and characterized by increased MAFG activation, GM-CSF signaling and pro-inflammatory pathway activity. The authors demonstrated that this population was present in 12 out of 20 MS patients and was strongly expanded in MS compared to controls. The same approach was used to validate that an anti-inflammatory LAMP1^+^TRAIL^+^ astrocyte subpopulation that was reduced in EAE compared to naïve mice, was also downregulated in MS (Sanmarco et al., 2021). It is notable that the 48 human samples used in these studies yielded only a total of 9,700 astrocyte nuclei, averaging 200 astrocyte nuclei per sample. This further illustrates that capture of astrocytes/astrocyte nuclei from human brain is sparse and that the analysis is limited by the low number of astrocyte representation.

There is currently a limited understanding of how isolation and purification protocols may change the nuclear yield of various CNS cellular constituents (Box et al., 2020; Denisenko et al., 2020; Ding et al., 2020). New sample preparation protocols have to be designed to address the structural and metabolic vulnerabilities of different nuclei from different brain regions. In addition, standard FACS technology relies on increased pressures (10–70 psi) for hydrodynamic focusing, and application of high voltage charges to the sorted sample path, which inflicts substantial damage on nuclei and may introduce sampling bias by depleting sensitive nuclear populations. Microfluidic sorters have become a mature technology that operates on atmospheric pressure and uses mechanical valves (Utharala et al., 2018; Berlanda et al., 2021). The reduced for electromechanical manipulation greatly aids the preservation of nuclei and the selective enrichment of sensitive and rare nuclei.

Moreover, new genomic protocols and high-throughput strategies are constantly emerging. Since their inception, 3-prime-based scRNA-seq protocols (Ramsköld et al., 2012; Macosko et al., 2015) have improved their sensitivity from initially 5–10 to 20% of RNA molecules captured per cell. Similarly, Smart-seq protocols have doubled their sensitivity from 30–40 to 70% (Smart-seq3), allowing for collection of the majority of transcripts, including isoforms, which ultimately improves the ability to identify biologically meaningful cell clusters (Hagemann-Jensen et al., 2020).

Finally, multi-omics approaches that combine single-cell transcriptomics with epigenetic mapping, proteomics and lineage tracing (Stoeckius et al., 2018; Gaublomme et al., 2019), provide ever-increasing molecular details of cellular states (Ludwig et al., 2019). These details can be further integrated with spatial transcriptomics which provides additional information such as phenotype localization within a given microenvironment and cell-to-cell interactions (Giesen et al., 2014; Liu et al., 2020). Applied to MS, these methodological advances will eventually provide a more complete picture of the cellular constituents that drive MS pathology, including rare and underrepresented cell types.

## Resolution of Astrocyte Heterogeneity With Highly Multiplexed Imaging

An additional important aspect of defining cell populations is to determine their spatial organization within a tissue environment such as MS lesions, including their spatial interactions and the environmental cues that drive their specific expression profiles. Moreover, the spatial resolution of phenotypes that have been determined by single cell genomics, will confirm and/or further improve the phenotypic separation. As with single cell genomics, substantial progress has been made in recent years with multiplexed spatial profiling of RNA and proteins in tissue.

### Spatial RNA Profiling

Technologies to quantify single-cell RNA levels in spatial context have been rapidly evolving. SmFISH and sequential FISH (seqFISH) approaches use fluorescence-labeled small oligonucleotides to probe single mRNA transcripts. SeqFISH uses multiple rounds of hybridization and has been shown to be scalable to the genome level *in vitro* (Eng et al., 2017). SmFISH has been used to define astrocyte subpopulations in different CNS regions (Batiuk et al., 2020; Bayraktar et al., 2020) and to map snRNA-seq-derived phenotypes onto MS lesion tissue (Jäkel et al., 2019; Schirmer et al., 2019). A further development that combines super-resolution microscopy with FISH, termed seqFISH+, achieves, in theory, multiplexing of >20,000 genes in single cells with high accuracy and sub-diffraction-limit resolution (Eng et al., 2019).

Additionally, a method termed deterministic barcoding in tissue for spatial omics sequencing (DBiT-seq) delivers DNA barcodes to tissue at a specific location via a microfluidics platform and sequences the spatially barcoded mRNA and proteins (Liu et al., 2020). Under ideal conditions, DBiT-seq is capable of spatially profiling thousands of mRNAs with next generation sequencing that can be co-mapped with proteins, albeit not at single-cell resolution. These technologies target the whole transcriptome but are currently not as robust as snRNA-seq with lower gene coverage and reading depth.

### Spatial Protein Profiling With Highly Multiplexed Imaging

Highly multiplexed imaging has been driven by imaging mass cytometry (IMC), a technology that, like CyTOF, uses antibodies conjugated to lanthanide isotopes (Giesen et al., 2014; Baharlou et al., 2019). Tissue sections are simultaneously labeled with up to one hundred antibodies, laser-ablated at high resolution and followed by time-of-flight mass spectrometry. IMC has several draw-backs that include the need for comprehensive validation and optimization of antibodies, high cost of antibody conjugation and the ablation/mass spectrometry procedure, lack of an amplification step, which reduces sensitivity for low-abundance proteins, and a standard magnification of only 16×, which may provide insufficient resolution. A low tech approach to highly multiplexed imaging is serial immunofluorescence staining of tissue sections with repetitive cycles of immunolabeling, scanning and antibody removal, termed iterative indirect immunofluorescent imaging (4i) (Gut et al., 2018). Similar to classical immunofluorescence labeling, this approach uses off-the-shelf antibodies, and allows for amplifications steps with secondary antibodies, high magnification imaging and acquisition of large tissue areas with a scanning microscope. The number of cycles is limited by decreasing tissue integrity. In our experience, the multiplexed fluorescent signals are quantitatively reproducible over at least 20 cycles without detectable loss of antigen, increase in background or tissue distortion. Per round, typically 2–4 antibodies can be applied, which allows for staining with a total of 40+ markers. Currently, a microfluidics-assisted version of 4i is being developed that automates most of this method and presumably better preserves tissue integrity. Other multiplexed protein imaging methods, such as nanostring (Decalf et al., 2019; Merritt et al., 2020) and CODEX (Schürch et al., 2020), rely on barcoding of antibodies with unique oligonucleotide sequences. These technologies allow for a one-time antibody application, followed by multiple rounds of binding of unique oligonucleotide reporters, each with a spectrally distinct dye, to assay the corresponding antibody barcodes.

### Computational Analysis

The different multiplexed imaging methods all require computational pipelines that include image registration (if multiple scans have been acquired), segmentation of cell types of interest with workflows such as ilastik pixel classification (Berg et al., 2019) and CellProfiler (Carpenter et al., 2006), and extraction of single cell information for cellular phenotype clustering that are visualized with dimensionality reduction methods such as principal component analysis (PCA) and *t*-distributed stochastic neighbor embedding (tSNE) (Amir et al., 2013), as with scRNA-seq or snRNA-seq data. The additional spatial data are analyzed with computational pipelines [e.g., histoCAT (Schapiro et al., 2017)] to localize cellular subpopulations within the tissue environment, to identify spatial interactions and to enrich phenotype transition trajectories for spatial context (Moon et al., 2019). Some of these tools are particularly relevant for tissues with complex cell morphologies such as CNS, where spatial analysis can focus separately on the cell body and peripheral processes. As highly multiplexed imaging methods evolve and are more broadly applied, new computational approaches for studying spatial patterns of cellular and molecular organization are emerging. This includes spatial variance component analysis (SVCA) (Arnol et al., 2019), which uses the marker profiles and spatial coordinates of each cell, to quantify the sources of variation for marker expression, such as cell-to-cell interactions, and intrinsic and environmental effects.

### Multiplexed Analysis of Glial Cells in MS Lesions

We have applied IMC to acute MS lesion tissue to investigate the landscape of myeloid and astrocyte phenotypes in acute and chronic active MS lesions (Park et al., 2019). In this study, we identified five astrocyte subtypes and six myeloid cell subpopulations based on expression patterns of 13 glial activation markers. We found that the different glial subpopulations localized to different lesional zones and exhibited subtype-specific spatial interactions. Moreover, we were able to elucidate astrocyte and microglia phenotypic transitions and quantify the effects of cell-intrinsic factors vs cell-to-cell interactions on marker expression in individual cells (Park et al., 2019). In a separate study of acute MS lesions, we were able to demonstrate the activation patterns of lymphocytes and microglia determined in previous MS studies. In particular, we were able to discriminate between the different source of demyelinating macrophages (microglia vs blood-derived macrophages) and to segregate different B and T cell phenotypes (Ramaglia et al., 2019). These studies demonstrate that highly multiplexed imaging, in conjunction with a computational analysis pipeline, provides a wealth of insight into the functional states and spatial organization of glial cells in MS lesions that are not accessible with standard histology.

## Summary of Single Cell Approaches to Determine Astrocyte Heterogeneity

**FIGURE 1:**
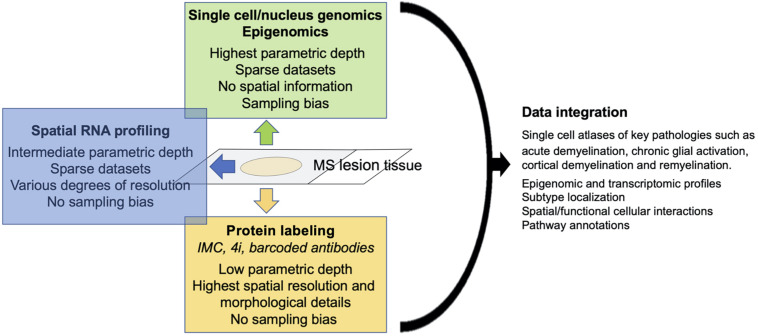
Schematic of strengths/weaknesses of single cell technologies and data output.

Single cell genomics, spatial mRNA profiling and highly multiplexed (protein) imaging offer different advantages and disadvantages. While both single cell genomics and spatial mRNA profiling target the whole transcriptome; RNA-seq is superior in isolated cells/nuclei compared to tissue with regard to gene coverage and sequencing depth. In contrast, highly multiplexed protein imaging has a parametric depth of only dozens to perhaps hundreds of markers, but allows for precise outlining of cells with complex, irregular morphologies such as astrocytes, which will improve the spatial analysis of individual cells. Although it is implicitly assumed that mRNA expression correlates with protein expression, this correlation varies substantially with different gene classes (Koussounadis et al., 2015), making it desirable to confirm RNA expression at a protein level, at least for key markers (Figure 1). Finally, single-cell gene expression matrix typically contains excessive zero entries (Yuan et al., 2017), resulting in sparse data sets, particularly in underrepresented cell types such as astrocytes. We observed that several key markers of astrocytes and microglia activation that were readily detectable with multiplexed imaging, were absent in single-cell transcriptomic datasets from MS lesions. Of note, spatial RNA transcriptomics and multiplexed protein profiling are not believed to lead to underrepresentation of specific cell types such as astrocytes and microglia. The technology is moving rapidly toward development of spatial multi-omics that incorporate mRNA profiling with protein profiling and epigenomics, as it is now possible with DBiT-seq (Liu et al., 2020).

## Conclusion

With optimized protocols to capture nuclei from astrocytes and other under-represented CNS cell types such as microglia, improvements in single cell genomics and multi-omics integration will allow generation of complete single-cell atlases of healthy CNS and neurological diseases. These atlases will include epigenomic and transcriptomic profiles, subpopulation localization, spatial and functional (receptor-ligand) cellular interactions and pathway annotations. Applied to carefully characterized MS tissue, this will identify the constituent subpopulations and cellular interactomes that are specific to key pathological processes such as acute demyelination, chronic glial activation in progressive MS, and remyelination. This comprehensive approach will identify therapeutic novel targets to ameliorate disease progression and to promote neurorepair.

## Author Contributions

CL, MS, MM, SB, ST, and DP wrote the manuscript. All authors contributed to manuscript revision, read, and approved the submitted version.

## Conflict of Interest

The authors declare that the research was conducted in the absence of any commercial or financial relationships that could be construed as a potential conflict of interest.

## Publisher’s Note

All claims expressed in this article are solely those of the authors and do not necessarily represent those of their affiliated organizations, or those of the publisher, the editors and the reviewers. Any product that may be evaluated in this article, or claim that may be made by its manufacturer, is not guaranteed or endorsed by the publisher.
